# The conundrum of parathyroidectomy vs cinacalcet for treatment of post-transplant hyperparathyroidism

**DOI:** 10.1590/2175-8239-JBN-2020-0107

**Published:** 2020-08-24

**Authors:** Rima Patel, Rowena Delos Santos

**Affiliations:** 1 Washington University School of Medicine, Department of Medicine, Division of Nephrology, United States.

Persistent hyperparathyroidism in renal transplant patients has been associated with increased mortality, increased risk of allograft loss, and increased risk of fractures and bone disease.^1^^,^ 2 The treatment of persistent hyperparathyroidism following kidney transplant remains a controversial one for transplant providers. There are differing opinions on whether cinacalcet or parathyroidectomy leads to better patient outcomes. Thus, the goal of any treatment would be not only to improve laboratory findings, but also to improve or stabilize graft outcomes, reduce mortality, and improve bone health, and should be studied when treatments are being evaluated.

In this issue of *Brazilian Journal of Nephrology*, Rivelli *et al.* 3 retrospectively examined total parathyroidectomy with implantation of a small portion of a parathyroid gland into the deltoid versus cinacalcet use for the treatment of severe persistent hyperparathyroidism post kidney transplant. The parameters evaluated include changes in serum calcium, phosphorus, PTH, and alkaline phosphatase levels over the course of 12 months of intervention. Their data indicate that patients who underwent parathyroidectomy had improvement in their hypercalcemia and hypophosphatemia and elevated PTH faster and more effectively than those receiving cinacalcet. However those who underwent parathyroidectomy also had decreased renal function at the end of the 12-month follow-up period compared to those in the cinacalcet group, though the authors do show that the parathyroidectomy group had slightly worse renal function at the start of the study.

A retrospective analysis by Tseng et al.^4^ echoed the findings of reduced renal function at 12 months, however showed renal recovery at 15 months compared to a cohort group of renal transplant recipients who did not undergo parathyroidectomy. Following the participants in the Rivelli study for a longer time period could potentially yield similar results. Additionally, a retrospective analysis by Kandil et al.^5^ also found decreased GFR in patients with parathyroidectomy but showed no change in 3-year graft survival. The mechanism for this change in renal function was postulated to be due to loss of vasodilatory effects on pre-glomerular vessels and vasoconstriction on efferent arterioles. The worsening GFR was thought to be due to reversal of glomerular hyperfiltration and not necessarily due to worsening renal function as evidenced by similar 3-year graft survival. In a randomized open label study by Cruzado et al^6^ comparing cinacalcet and parathyroidectomy, there was no significant difference in renal function between the groups post treatment.

Though the cinacalcet group in the Rivelli study were slower to achieve improvement in their serum calcium levels, the study also shows that 95% of those patients did not achieve normalization of their PTH. A randomized study comparing cinacalcet to placebo by Evenepoel at al.^7^ has shown that though cinacalcet can improve biochemical parameters, as confirmed by the present study, there was no significant difference between cinacalcet and placebo in bone mineral density at the femoral neck by DEXA scan. Of note, this study was a 52-week study, and upon withdrawal of cinacalcet, PTH rebounded to a mean of 234 pg/mL in the cinacalcet group vs 277 pg/mL in the placebo group. This suggests that for patients with persistent and severe hyperparathyroidism post transplant, there was very limited, if any, regression of parathyroid hyperplasia compared to cinacalcet treatment. While one can argue that involution of the gland requires time, only a study with much longer follow-up, such as 5 to 10 years, would be able to help elucidate this question.

A study by Borchhardt et al.^8^ suggests a risk of adynamic bone disease with use of cinacalcet in transplant patients. A randomized control trial done by Cruzado et al^6^ comparing cinacalcet and parathyroidectomy indicates that patients in the parathyroidectomy group had benefit in bone mineral density while the cinacalcet group did not. Therefore, though both methods improve laboratory parameters there is evidence to suggest that parathyroidectomy may improve overall bone health.

There were limitations in the present study including the significant era differences between the two groups. These periods had different patters of induction and maintenance immunosuppression prescriptions and hence differed in renal function and allograft survival. Overall, there is a paucity of data for clinical outcomes in this cohort of patients and much of the data that currently exists similarly have small sample sizes. Future research should include more indicators of bone health, cardiovascular health, mortality, and allograft survival in order to optimize care of transplant patients with post transplant persistent hyperparathyroidism. The potential for worsening allograft function seen in this study as well as others is concerning and larger randomized control trials are indicated to evaluate this in the future. Other studies looking at the long-term effects of prolonged cinacalcet use on kidney allograft and bone health would be valuable.

Given the current data, at our center, we recommend that patients with significantly elevated PTH (defined as > 1000 pg/mL without calcimimetic use, or 500 pg/mL with calcimimetic use) have subtotal parathyroidectomy prior to transplant. We monitor calcium, phosphorus, PTH, and alkaline phosphatase over the first year and medically manage without the use of cinacalcet due to the possibility of resolution of secondary hyperparathyroidism after transplant. However, patients with persistent hyperparathyroidism after the first year post transplant are referred for subtotal parathyroidectomy. If patients do not want to undergo surgery or are at high risk for surgery, then they are treated with cinacalcet.
